# Are Steroid Hormones Dysregulated in Autistic Girls?

**DOI:** 10.3390/diseases8010006

**Published:** 2020-03-14

**Authors:** Benedikt Andreas Gasser, Johann Kurz, Bernhard Dick, Markus Georg Mohaupt

**Affiliations:** 1 Department of Clinical Research, University of Bern, 3010 Berne, Switzerland; bernhard.dick@gmx.ch; 2 Teaching Hospital Internal Medicine, Lindenhofgruppe, 3006 Berne, Switzerland; john@a1.net (J.K.); markus.mohaupt@lindenhofgruppe.ch (M.G.M.)

**Keywords:** gas chromatography-mass spectrometry, cholesterol, 21-hydroxylase

## Abstract

Evidence of altered cholesterol and steroid hormones in autism is increasing. However, as boys are more often affected, evidence mainly relates to autistic males, whereas evidence for affected autistic girls is sparse. Therefore, a comprehensive gas chromatography mass spectrometry-based steroid hormone metabolite analysis was conducted from autistic girls. Results show increased levels of several steroid hormones, especially in the class of androgens in autistic girls such as testosterone or androstenediol. The increase of the majority of steroid hormones in autistic girls is probably best explained multifactorially by a higher substrate provision in line with the previously developed cholesterol hypothesis of autism.

## 1. Introduction

**Figure 1:**
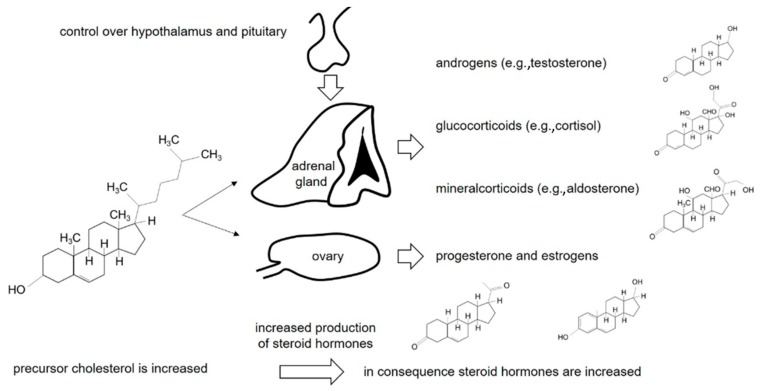
The potential mechanism of increased steroid hormones in affected autistic individuals. The increased availability of the precursor cholesterol yields control of the hypothalamus pituitary adrenal gland (HPA) to the corticotropin-releasing hormone (CRH)-adrenocorticotropic hormone (ACTH) system and ovary and leads to increased metabolites, such as testosterone or cortisol, which alter critical developmental steps leading to autistic disorders.

Several times, autism has been associated with altered steroid hormones and, in consequence, the hypothalamic pituitary adrenal (HPA) axis [1,2,3,4,5]. One hypothesis suggests increased substrate availability (cholesterol) yielding increased steroid hormones [6,7]. Alterations of these hormones subsequently impact the development of autism [6,7]. Furthermore, as autism is diagnosed more often in boys than in girls, long speculation and controversy about the role of “*the male brain*” and sex hormones (all of them biochemical steroids) have been evoked [8,9,10,11,12]. In addition, autism has also recently been linked to stress, altered stress hormone levels [13] and vitamin D insufficiency which is another derivative of cholesterol [14,15,16] (Figure 1).

From a more comprehensive standpoint, some studies suggest the involvement of the corticotropin-releasing hormone (CRH)-adrenocorticotropic hormone (ACTH) system with additional alterations of adrenal gland metabolites in autistic disorders [5,17,18,19,20,21] (Figure 1). Differences in affected autistic individuals versus healthy controls were found at the level of the hypothalamus [2,13], at the level of the pituitary gland [5,17,18,22,23,24] and at the level of adrenal gland [4,22,25,26]. An increase in cortisol was found in autistic individuals [1,17] and, in addition, cortisol response to stress stimuli was altered [4]. Several times, increased androgens were associated with autistic children, adolescents and adults [25,26,27,28,29] (Figure 1). Supporting these findings, salivary levels of steroids in prepubertal autistic male and female children and levels of several androgens were increased [30], and a positive correlation between testosterone and the severity of autism was subsequently implied [31]. For mineralocorticoids, therapeutic recommendations were made with substances directly inhibiting a mineralocorticoid receptor (spironolactone), whereby positive effects were described for autistic symptoms by treatment [32,33].

However, this evidence is mainly derived from males, whereas for women, evidence is sparse despite some hints of the relevance of involvement of the two typical female hormones—progesterone and oestrogen [11,25,26,34,35]. This being the case, an understanding of a potential alteration of these hormones in autistic girls might provide further clues concerning the mechanisms that drive this neurodevelopmental disorder. In particular, potential sex-dependent alterations in steroid hormones might provide further evidence related to the underlying nosology of autism. This begs the question of whether steroid hormones in affected autistic girls are increased compared to those in unaffected girls. As a hypothesis with potential falsification, it shall be stated that there is no difference between affected and unaffected autistic individuals [36].

## 2. Materials and Methods

### 2.1. Participants

Sixteen autistic girls (BMI 17.4 ± 2.8; average age 14.3 + 4.2 years) and a matched control cohort for age, weight and height (BMI 16.8 ± 2.4; average age 14.4 ± 4 years). No significant difference for BMI or average age could be detected between the groups of affected and unaffected girls.

### 2.2. Study Design

Autistic and control girls were recruited from the area of Leipzig (Austria). Enrolment took place from mid-2009 to mid-2012. All participants were Caucasians. Participants were excluded if they had a history of liver diseases, renal or endocrine disorders, a current infection, or fever. Intellectual disability or behavioural disorders were exclusion criteria only for the control group but were allowed as comorbid conditions in the autistic group, whereby one girl had to be categorized as intellectually disable. The diagnosis was given in the first years of the children’s lives according to the diagnostic criteria of the DSM-IV and was cross-checked by experienced clinicians (i.e., medical doctors and/or psychologists) during enrolment of the study. All procedures performed in the studies involving human participants were in accordance with the ethical standards of the institutional and/or national research committee and with the 1964 Helsinki Declaration and its later amendments or with comparable ethical standards. The study was approved by the governmental ethics board of Graz, Austria, and registered at ClinicalTrials.gov. Involvement in the study was voluntary and not compensated. After the study procedures were fully explained, the parents of the participants read and signed informed consent forms. All authors declare that they do not have any conflicts of interest.

### 2.3. Methods

Analysis of urinary steroids was conducted via gas chromatography-mass spectrometry. Urine samples were taken in the morning after breakfast (the first urine of the day, not later than 9 a.m.). Urine sample preparation comprised pre-extraction, enzymatic hydrolysis, extraction from the hydrolysis mixture, derivatization and gel filtration. The recovery standard was prepared by adding 2.5 µg of medroxyprogesterone to 1.5 mL of urine. The sample was extracted on a Sep-Pak C18 column (Waters Corp., Milford, MA, USA), dried, reconstituted in a 0.1 M acetate buffer, pH 4.6, and hydrolysed with a powdered Helix pomatia enzyme (12.5 mg; Sigma Chemical Co., St. Louis, MI, USA) and 12.5 µL of β-glucuronidase/arylsulfatase liquid enzyme (Roche Diagnostics, Rotkreuz, Switzerland). The resulting free steroids were extracted on a Sep-Pak C18 cartridge. A mixture of internal standards (2.5 µg each of 5α-androstane-3α, 17α-diol, stigmasterol, and cholesterol butyrate, and 0.15 µg of 3β5β-tetrahydroaldosterone) was added to this extract, and the sample was derivatised to form the methyloxime-trimethylsilylethers. Analyses were performed on a Hewlett Packard gas chromatograph 6890 (Hewlett Packard, Palo Alto, CA, USA) with a mass selective detector 5973 by selective ion monitoring (SIM). One characteristic ion was chosen for each compound measured. The derivatised samples were analysed during a temperature-programmed run (210–265 °C) over a 35 min period. The calibration standard consisted of a steroid mixture containing known quantities of all the steroid metabolites to be measured. Responses and retention times were recorded regularly. In each case, the ion peak was quantified against the internal stigmasterol standard. While using the abovementioned methods, we present in this work the 39 most important relevant metabolites of the steroid hormone synthesis pathways (Table 1 and Table 2). All steroid hormone metabolites were corrected for urinary creatinine excretion. Apparent enzyme activities were calculated as previously described by us and others [37,38,39].

### 2.4. Statistical Analysis

The mean and SEM (standard error of mean) of all metabolites were calculated. In order to analyse the distribution patterns of the measured values of each metabolite in autistic girls and control girls, Kolmogorov–Smirnov tests were conducted. If normality distribution was present, the differences between autistic and control children were analysed with two-tailed heteroscedastic *t*-tests. As for dehydroepiandrosterone, 17b-estradiol, 17-hydroxypregnanolon, pregnanediol, tetrahydrocorticosterone, 20a-dihydrocortisol, 18-hydroxycortisol, the hypothesis of normal distribution had to be rejected. Wilcoxon tests were conducted for the comparison of all autistic girls versus controls (Table 1). Due to the small sample sizes for the abovementioned metabolites, Mann–Whitney U tests were performed for prepubertal and post-pubertal analyses concerning the differences between autistic girls and controls (Supplementary Materials Table S1) [40,41,42]. To correct for multiple comparison, Bonferroni correction was performed. Differences were further quantified between autistic versus healthy subjects with the calculation of effect sizes by Cohen with pooled standard deviation and 95% confidence intervals if normal distribution was present [43,44]. For metabolites not showing a normal distribution, effect sizes were calculated according to Pallant [45]. Ratios of enzymes were calculated as indicated in Table 2 and differences of ratios were analysed with two-tailed paired *t*-tests. Calculations were performed with GraphPad Prism (GraphPad Software, Inc., La Jolla, CA, USA) and Microsoft Excel (Microsoft Inc., Redmond, WA, USA).

## 3. Results

Average concentrations in the urine of affected autistic girls versus matched unaffected controls are shown in Table 1. Mainly increased values of androgens can be detected in affected autistic girls. However, besides increased steroid hormone values, there were two significant decreased ones with the corticosterone metabolite 18-hydroxy-tetrahydrocompound A and the cortisol metabolite b-cortol weakening the argument of a potential hyperandrogenism and hypercortisolism, while indicating more broadly dysregulated steroid hormones. This seems further supported when correcting for multiple comparisons as proposed by others [25]. If corrected for the three main classes of steroid hormones (i.e., glucocorticoids, mineralocorticoids, and androgens) only testosterone and androstenediol remain significantly increased. The calculated effect sizes by Cohen remain impressive, and all androgen metabolites had a positive overall effect size of 0.6 (Cl −0.11,1.31) which has to be taxed as moderate to large.

Despite an already relatively small sample it was tried to control for pre versus post pubertal state as suggested by others (Supplementary Table S1) [40,41,42]. Here, autistic girls were divided in sub-13 years, indicating a prepubertal state, versus older than 13, indicating post-pubertal state [40,41,42]. The results become very vague probably also due to the very small sample size especially in prepubertal autistic girls (*n* = 5). However, even with Bonferroni correction for classes (glucocorticoids, mineralocorticoids, androgens), significant alterations remain in prepubertal autistic girls for 18-hydroxy-tetrahydrocompound A and b-cortol. In the post-pubertal autistic girls androstenediol, testosterone, tetrahydroaldosterone, 18-hydroxycortisol and cortisol are significantly increased.

In Table 2, the activities of enzymes calculated as ratios of metabolites are shown. Neither a 11-hydroxylase deficit, 17-hydroxylase deficit, nor a 11-beta hydroxylase can be identified in affected autistic children. However, worth mentioning is the highly significant ratio of cortisol to cortisone in line with an altered activity of 11-beta hydroxylase, increased substrate availability of cortisol and in consequence a hypercortisolism. Furthermore, a 21-hydroxylase deficit might be prevalent. Focusing on typical female hormones In contrast to androgens, no significantly increased metabolites were detected.

## 4. Discussion

The aim of the study was to analyse steroid metabolites in affected autistic girls. To conclude, increased values of several androgens, such as testosterone and glucocorticoids including cortisol, were detected for the whole sample of autistic girls versus healthy controls. A general increase of glucocorticoids would favour the premise of a dysregulation of the HPA axis, especially with regard to the CRH-ACTH system (Figure 1). Some studies already suggested an involvement of the corticotropin-releasing hormone (CRH)-adrenocorticotropic hormone (ACTH) system with additional alterations of adrenal gland metabolites in autistic disorders [4,5,17,18,19]. The regulation of proangiogenic factors was investigated in adrenocortical cells isolated from human foetal adrenal glands for a more comprehensive understanding [45,46,47]. The ACTH upregulates vascular-endothelial growth factor–A (VEGF-A) and increases mRNA encoding of angiopoietin 1, indicating that ACTH is the primary regulator of adrenal organ growth by stimulating VEGF and thus angiogenesis, allowing to link the hypothalamic–pituitary–adrenal axis (HPA axis) with adrenal gland development [46,47,48].

For enzyme activities, no 17-hydroxylase deficit, 11-hydroxylase deficit nor 11-beta hydroxylase deficit were detected. A 21-hydroxylase deficit seems prevalent which can be linked to findings related to genetic factors involved in androgen metabolism, such as CYP11B1, CYP17A1 and CYP19A1, that are relevant for androgen synthesis (e.g., via aromatase) and have been previously described [49,50,51,52]. Recent genomic advances have led to the discovery of diverse genetic loci linked to autistic disorders, including chromosomal aberrations [53,54], copy number variations [52,53] and both common and rare single nucleotide variations [55,56,57,58,59,60,61,62,63]. Consequently, to date, more than 330 candidate genes have been associated with autism susceptibility [63,64] Genes involved in synapse formation and brain circuitry are consistently found to be dysregulated in people with autism and the influence of steroid hormones was implied several times [49,63,64,65,66,67,68].

To sum up, we are far away from a complete understanding of the mechanisms and results are limited by the relatively small sample size and the fact that only single-point measurements were made. However, the alterations of steroid hormones show at least where potential further research might be aimed. To the best of our knowledge, these suggestions are not yet supported by an animal model nor by other clinical trials from girls. Larger longitudinal measurements of cohorts, which might give further hints concerning the underlying mechanisms, are necessary

## 5. Conclusions

The findings of an increase in some steroid hormones in affected autistic girls but not of enzymes metabolizing these would be in line with the premise of an increased substrate provision of cholesterol (cholesterol hypothesis of autism [7]).

These alterations would probably be best explained multifactorially by a higher substrate provision with disturbed feedback loops of the central nervous system by the hypothalamus–pituitary–adrenal axis and sex organs.

Furthermore, alterations of steroid hormones might be used as diagnostic markers in order to secure a clinical diagnosis of autism.

## Figures and Tables

**Table 1:** Encompassing steroid hormone metabolites of affected autistic girls versus individually pairwise matched controls (*n* = 16). The term Wilcoxon behind the metabolite indicates that Wilcoxon tests instead of two-sided heteroscedastic *t*-tests were performed.

Urinary Steroid Hormone Metabolites	Autistic Girls				Control Girls				95% CI	
(μg/mmol Creatinine)	Median	Mean	SEM	*p*-Value	Median	Mean	SEM	Effect Size	Lower Limit	Upper Limit
**Androgen-Metabolites**										
Androsterone	28.5	71.7	21.4	0.15	35.2	42.1	9.1	0.46	−0.25	1.16
Etiocholanolone	24.6	57.8	16.6	0.17	26.4	38.8	14.3	0.32	−0.39	1.00
Androstenediol	1.7	2.2	0.4	0.02	1.2	1.3	0.2	0.71	0.01	1.44
11-Oxo-Etiocholanolon	30.4	35.1	6.7	0.11	19.2	20.9	3.0	0.67	−0.04	1.39
11b-Hydroxy-Androsteron	29.5	36.6	5.5	0.08	24.4	22.1	3.2	0.77	0.09	1.53
11b-Hydroxy-Etiocholanolon	28.9	26.0	5.2	0.06	12.5	13.1	2.0	0.78	0.06	1.50
Dehydroepiandrosteron (Wilcoxon)	2.8	15.2	8.4	0.17	3.7	4.3	1.0	0.47		
5-Androstene-3b,17b-diol	3.4	6.8	2.6	0.10	1.8	2.2	0.4	0.62	−0.09	1.33
16a-Hydroxy-DHEA	8.2	57.6	36.9	0.20	6.0	8.0	1.5	0.48	−0.33	1.07
5-Androstene-3b,16a,17b-triol	8.1	36.8	16.8	0.12	7.2	8.6	1.9	0.59	−0.11	1.31
5-Pregnene-3b, 16a,17b-triol	4.4	11.6	4.4	0.70	6.8	9.7	1.7	0.14	−0.50	0.89
Testosterone	0.8	0.8	0.1	0.02	0.5	0.5	0.1	0.83	0.26	1.73
5a-Dihydrotestosteron	1.2	1.2	0.2	0.94	1.2	1.2	0.1	0.03	−0.51	0.87
**Oestrogen-Metabolites**										
Estriol	0.1	0.6	0.4	0.11	0.1	0.4	0.1	0.19	−0.60	0.78
17b-Estradiol (Wilcoxon)	0.046	0.1	0.0	0.10	0.1	0.2	0.1	−0.49		
**Progesterone-Metabolites**										
17-Hydroxypregnanolon (Wilcoxon)	2.7	5.5	1.4	0.33	3.3	3.5	0.6	0.47		
Pregnanediol (Wilcoxon)	11.6	18.9	4.6	0.50	10.8	12.7	2.4	0.44		
Pregnanetriol	18.4	33.0	9.2	0.17	19.3	20.5	3.5	0.46	−0.23	1.18
11-Oxo-Pregnanetriol (Wilcoxon)	1.0	1.2	0.2	0.99	1.1	1.4	0.4	−0.20		
**Aldosterone-Metabolites**										
Tetrahydroaldosterone	1.9	2.2	0.5	0.05	4.1	1.1	0.1	0.72	0.04	1.48
**Corticosterone-Metabolites**										
TetrahydroDOC	0.5	0.6	0.1	0.71	1.0	0.5	0.1	0.14	−0.50	0.89
Tetrahydrodehydrocorticosterone	9.5	10.2	1.6	0.71	0.4	9.4	1.2	0.14	−0.59	0.80
Tetrahydrocorticosterone (Wilcoxon)	9.3	9.2	1.0	0.4965	9.7	9.6	2.1	−0.07		
5a-Tetrahydrocorticosteron	18.6	18.9	2.8	0.43	8.2	24.8	6.7	−0.30	−1.00	0.40
18-Hydroxy-Tetrahydrocompound A (Wilcoxon)	1.1	1.9	0.6	0.04	24.8	7.1	2.1	−0.81		
**Cortisone**	9.0	10.2	0.9	0.70	3.9	10.9	1.5	−0.15	−0.89	0.50
**Cortisone-Metabolites**										
Tetrahydrocortisone	176.6	203.4	22.6	0.75	10.3	194.1	25.0	0.10	−0.60	0.79
a-Cortolon	65.2	70.2	7.7	0.23	194.1	56.4	7.0	0.48	−0.22	1.18
b-Cortolon	169.0	220.4	69.1	0.02	62.2	43.3	5.5	0.85	0.17	1.63
20a-Dihydrocortison	1.1	1.2	0.1	0.87	47.2	1.2	0.2	0.07	−0.69	0.69
20b-Dihydrocortison	3.4	3.7	0.5	0.49	1.2	3.2	0.5	0.29	−0.44	0.95
**Cortisol**	5.9	6.3	0.6	0.02	3.0	4.1	0.5	0.41	0.26	1.73
**Cortisol-Metabolites**										
Tetrahydrocortisol	61.9	68.4	8.5	0.62	4.7	61.5	9.0	0.21	−0.49	0.90
5a-Tetrahydrocortisol	56.7	77.0	16.1	0.54	52.4	96.7	23.7	-0.25	−0.94	0.45
a-Cortol	10.8	13.5	1.8	0.65	89.4	12.1	1.9	0.20	−0.55	0.84
b-Cortol	12.8	15.2	2.3	0.08	12.0	26.0	4.4	-0.75	−1.52	-0.08
20a-Dihydrocortisol (Wilcoxon)	2.3	3.2	0.6	0.40	3.2	3.6	0.7	-0.15		
6b-Hydroxycortisol	9.3	11.1	1.5	0.55	9.8	9.8	1.4	0.23	−0.47	0.92
18-Hydroxycortisol	27.0	35.4	4.8	0.02	19.9	21.3	3.1	0.83	−0.47	0.92
**Total**										
Total Androgens	150.0	359.4	105.1	0.08	122.0	172.8	29.8	0.60	−0.11	1.31
Total of Cortisol and Cortisone	610.9	740.9	101.3	0.13	605.2	552.1	75.9	0.53	−0.17	1.24
Total - All Measured Metabolites	982.2	1206.3	170.2	0.08	835.2	814.4	96.3	0.70	0.03	1.47

**Table 2:** Enzyme activities in affected prepubertal autistic girls (*n* = 5) and post-pubertal autistic girls (*n* = 11) versus matched controls. Tetrahydroaldosterone (THADLO), 18-Hydroxy-tetrahdrocompound (18OHTHA-THALDO), Pregnanolone (HP), 17-Hydroxypregnanolon (17-HP), TetrahydroDOC (THDOC), Tetrahydrodehydro-corticosterone (THA), Tetrahydrocorticosterone (THB), 5a- Tetrahydrocorticosterone (5a-THB), 5a-Tetrahydrocortisol (5a-THF), Tetrahydrosubstance S (THS), 11-Oxo-Pregnanetriol (PT’ONE), Tetrahydrocortisone (THE), Tetrahydrocortisol (THF), 5a-Tetrahydrocortisol (5a-THF).

	Autistic Girls			Control Girls	
	Mean	SEM	*p*-Value	Mean	SEM
**18OHTHA/THALDO**	1.175	0.399	0.014	1.595	0.419
**21-Hydroxylase Deficit**					
17HP/(THE + THF + 5aTHF)	0.017	0.003	0.026	0.010	0.002
PT/(THE + THF + 5aTHF)	0.098	0.016	0.064	0.073	0.013
100 × PT’ONE/(THE + THF + 5aTHF)	0.358	0.043	0.644	0.404	0.099
**17-Hydroxylase Deficit**					
(THA + THB + 5aTHB)/(THE + THF + 5aTHF)	0.256	0.012	0.407	0.287	0.021
100 × THDOC/(THE + THF + 5aTHF)	0.257	0.069	0.157	0.145	0.018
**11-Hydroxylase Deficit**					
100 × THS/(THE + THF + 5aTHF)	1.682	0.152	0.330	1.864	0.151
100 × THDOC/(THE + THF + 5aTHF)	0.257	0.069	0.157	0.145	0.018
**11-Beta Hydroxylase**					
F/E	0.608	0.035	0.002	0.445	0.026
(THF + 5aTHF)/THE	0.642	0.058	0.290	0.044	0.004
(F + E)/THE + THF + 5aTHF	0.057	0.005	0.788	0.044	0.004

## Supplementary Materials

The following are available online at https://www.mdpi.com/2079-9721/8/1/6/s1, Figure S1: Encompassing steroid hormone metabolites of affected prepubertal autistic girls (n = 5) and postbuertal autistic girls (n = 11) versus individually pairwise matched controls. The term Mann-Whitney U behind a metabolite indicates that these tests instead of two-sided heteroscedastic t-tests were performed.
