# Supplementary material for: Are Steroid Hormones Dysregulated in Autistic Girls?
Source: Diseases. 2020 Mar 14;8(1):6. doi: 10.3390/diseases8010006 (PMC7151154; doi:10.3390/diseases8010006)
Supplement: Supplementary file 1 [file diseases-08-00006-s001.pdf]

Article

Are Steroid Hormones Dysregulated in Autistic Girls?

Benedikt Andreas Gasser [1,*], Johann Kurz [2], Bernhard Dick [1] and Markus Georg Mohaupt [2]

[1] Department of Clinical Research, University of Bern, 3010 Berne, Switzerland; bernhard.dick@gmx.ch
[2] Teaching Hospital Internal Medicine, Lindenhofgruppe, 3006 Berne, Switzerland; john@a1.net (J.K.), markus.mohaupt@lindenhofgruppe.ch (M.G.M.)
* Correspondence: gasser@pyl.unibe.ch

Supplementary Table 1: Encompassing steroid hormone metabolites of affected prepubertal autistic girls (n=5) and postbuertal autistic girls (n=11) versus individually pairwise matched controls. The term Mann-Whitney U behind a metabolite indicates that these tests instead of two-sided heteroscedastic t-tests were performed.

	**prepubertal**										**postpubertal**									
Urinary steroid hormone metabolites	autistic girls				control girls				95%-CI		autistic girls				control girls				95%-CI	
[µg/mmol creatinine]	Median	Mean	SEM	p-value	Median	Mean	SEM	effect size	lower limit	upper limit	Median	Mean	SEM	p-value	Median	Mean	SEM	effect size	lower limit	upper limit
**Androgen-Metabolites**																				
Androsteron	16.26	19.90	4.35	0.63	15.49	29.20	15.65	-0.38	-1.60	0.91	66.22	97.54	29.05	0.09	49.68	48.49	11.16	0.68	-0.20	1.53
Etiocholanolon	16.19	16.74	2.72	0.39	6.80	11.17	4.33	0.68	-0.64	1.91	41.09	78.35	22.35	0.22	29.67	52.59	20.16	0.39	-0.48	1.21
Androstenediol	1.48	1.48	0.09	0.42	0.95	1.18	0.28	0.63	-0.93	1.57	2.20	2.62	0.59	0.03	1.23	1.37	0.24	0.81	-0.08	1.66
11-Oxo-Etiocholanolon	36.42	34.55	7.89	0.41	25.20	23.39	5.40	0.72	-0.51	2.09	30.07	35.43	9.56	0.20	19.18	19.71	3.80	0.66	-0.19	1.53
11b-Hydroxy-Androsteron	25.35	27.17	2.49	0.84	26.61	27.91	2.78	-0.13	-1.24	1.24	36.63	41.30	7.91	0.07	18.04	19.24	4.37	0.97	0.15	1.94
11b-Hydroxy-Etiocholanolon	30.78	23.55	6.40	0.44	16.82	16.09	4.07	0.62	-1.55	0.94	28.54	27.18	7.36	0.10	11.35	11.55	2.28	0.84	0.01	1.77
Dehydroepiandrosteron (Mann-Whitney)	1.45	2.56	1.12	0.40	2.25	3.24	1.42	-0.26			5.07	21.53	12.30	0.35	4.15	4.79	1.28	0.20		
5-Androstene-3b,17b-diol	0.87	1.23	0.34	0.98	0.63	1.21	0.61	0.02	-1.20	1.28	5.35	9.65	3.58	0.06	2.36	2.74	0.55	0.80	-0.08	1.67
16a-Hydroxy-DHEA	2.52	3.55	1.27	0.58	3.54	5.79	3.05	-0.44	-1.69	0.82	19.62	84.59	54.17	0.19	10.47	9.04	1.73	0.61	-0.27	1.44
5-Androstene-3b,16a,17b-triol	3.76	4.85	1.77	0.99	1.87	4.82	2.52	0.01	-1.24	1.24	13.13	52.72	23.96	0.10	10.78	10.43	2.33	0.75	-0.13	1.61
5-Pregnene-3b, 16a,17b-triol	2.19	2.78	0.63	0.14	6.23	8.20	2.72	-1.07	-2.62	0.14	8.86	15.95	6.22	0.39	13.08	10.49	2.15	0.37	-0.50	1.19
Testosteron	0.60	0.56	0.10	0.72	0.51	0.50	0.07	0.34	-0.92	1.58	0.98	0.93	0.12	0.02	0.57	0.52	0.11	1.01	0.19	2.00
5a-Dihydrotestosteron	1.47	1.44	0.23	0.52	1.46	1.36	0.13	0.21	-1.04	1.45	0.96	1.15	0.22	0.95	1.11	1.17	0.20	-0.03	-0.86	0.81
**Oestrogen-Metabolites**																				
Estriol	0.11	0.13	0.05	0.50	0.06	0.06	0.03	0.64	-0.33	2.34	0.22	0.80	0.56	0.41	0.11	0.39	0.15	0.31	-0.47	1.22
17b-Estradiol (Mann-Whitney)	0.04	0.04	0.01	0.58	0.01	0.02	0.01	0.20			0.06	0.12	0.06	0.10	0.10	0.22	0.08	0.30		

**Progesteron-Metabolites**																				
17-Hydroxypregnanolon (Mann-Whitney)	2.53	3.86	1.25	0.83	2.99	2.48	0.47	0.07			5.08	6.37	1.99	0.62	3.52	4.02	0.91	0.09	-0.40	1.29
Pregnanediol (Mann-Whitney)	11.57	13.14	1.84	0.83	11.71	11.81	2.12	0.07			13.23	21.84	6.74	0.38	10.83	13.13	3.49	0.19	-0.37	1.33
Pregnanetriol	17.58	15.76	1.97	0.83	13.82	15.45	2.77	0.06	-1.20	1.28	27.48	41.63	13.12	0.18	20.46	22.97	4.94	0.58	-0.30	1.40
11-Oxo-Pregnanetriol (Mann-Whitney)	1.22	1.44	0.47	0.83	0.98	0.97	0.08	0.07			0.94	1.09	0.23	0.73	1.19	1.68	0.56	-0.07	-1.27	0.42
**Aldosteron-Metabolites**																				
Tetrahydroaldosterone	2.70	3.53	1.39	0.25	1.69	1.48	0.17	0.87	0.35	3.46	1.80	1.55	0.15	0.02	0.96	0.95	0.12	1.14	0.38	2.24
**Corticosteron-Metabolites**																				
TetrahydroDOC	0.52	0.65	0.17	0.69	0.46	0.85	0.40	-0.31	-1.54	0.96	0.44	0.55	0.13	0.20	0.32	0.35	0.06	0.61	-0.24	1.48
Tetrahydrodehydrocorticosteron	9.57	13.33	3.94	0.85	11.98	12.39	1.08	0.15	-1.11	1.37	8.53	8.62	1.33	0.71	6.94	7.97	1.46	0.15	-0.69	0.99
Tetrahydrocorticosteron (Mann-Whitney)	7.84	9.40	1.72	0.84	8.98	13.59	5.55	-0.07			10.12	9.05	1.36	0.24	5.92	7.65	1.53	0.25	-0.55	1.13
5a-Tetrahydrocorticosteron	20.80	22.80	2.78	0.33	26.56	42.92	16.99	-0.72	-2.01	0.57	13.71	17.00	3.92	0.81	15.91	15.70	3.44	0.11	-0.67	1.00
18-Hydroxy-tetrahydrocompound A	1.07	0.96	0.42	0.01	8.54	10.39	2.69	-0.79	-3.79	-0.53	1.18	2.32	0.89	0.31	3.30	5.42	2.74	0.30	-1.31	0.39
**Cortisone**	8.35	11.20	2.17	0.26	16.48	16.15	2.49	-0.89	-2.21	0.42	9.88	9.69	0.97	0.16	7.92	8.30	1.25	0.40	-0.56	1.12
**Cortisone-Metabolites**																				
Tetrahydrocortisone	180.55	231.75	35.66	0.28	257.65	271.74	29.02	-0.56	-1.71	0.81	165.80	189.17	29.00	0.41	114.61	155.21	27.74	0.38	-0.48	1.20
a-Cortolon	65.22	75.95	13.09	0.83	83.04	79.21	5.61	-0.15	-1.40	1.09	64.31	67.34	9.88	0.16	36.19	45.03	8.04	0.74	-0.12	1.61
b-Cortolon	199.89	350.16	176.67	0.17	58.30	62.41	7.09	0.95	0.80	4.32	153.31	155.55	52.97	0.05	28.51	33.81	5.46	0.92	0.08	1.86
20a-Dihydrocortison	0.94	1.26	0.28	0.34	1.46	1.85	0.42	-0.73	-2.08	0.52	1.10	1.23	0.18	0.18	0.79	0.87	0.12	0.71	-0.04	1.71
20b-Dihydrocortison	3.31	4.01	0.80	0.54	4.60	4.96	0.79	-0.54	-1.80	0.73	3.46	3.61	0.67	0.16	2.28	2.33	0.37	0.72	-0.14	1.59
**Cortisol**	4.90	6.35	1.38	0.73	5.96	7.37	2.03	-0.28	-1.50	0.99	6.13	6.11	0.80	0.03	4.30	3.79	0.50	0.98	0.16	1.95
**Cortisol-Metabolites**																				
Tetrahydrocortisol	60.30	60.68	7.75	0.40	67.46	75.96	15.72	-0.56	-1.73	0.79	70.33	72.26	12.31	0.35	39.23	54.27	10.74	0.49	-0.38	1.32
5a-Tetrahydrocortisol	63.58	68.49	8.61	0.15	117.39	170.54	55.14	-1.03	-2.47	0.25	54.24	81.22	24.15	0.52	71.13	59.80	13.48	0.35	-0.51	1.18
a-Cortol	10.58	12.13	1.79	0.22	16.02	17.89	3.86	-0.82	-2.08	0.51	11.76	14.16	2.65	0.21	10.95	9.20	1.38	0.71	-0.19	1.54
b-Cortol	11.12	12.45	1.48	0.01	38.14	41.32	6.22	-1.61	-4.67	-0.97	13.77	16.58	3.41	0.21	10.83	18.37	4.06	-0.15	-1.00	0.67
20a-Dihydrocortisol (Mann-Whitney)	2.30	3.48	1.09	0.21	3.46	5.31	1.99	-0.40			2.35	3.05	0.82	0.69	3.06	2.72	0.25	-0.27		
6b-Hydroxycortisol	8.27	12.48	4.24	1.00	10.49	12.50	2.56	0.00	-1.24	1.24	9.61	10.34	1.19	0.37	6.34	8.39	1.60	0.44	-0.41	1.28
18-Hydroxycortisol	25.38	42.97	13.44	0.38	32.16	30.73	4.86	0.55	-0.54	2.05	27.89	31.60	3.01	0.02	12.01	16.52	3.05	1.24	0.53	2.44
**Total**																				
total Androgens	138.19	140.38	3.63	0.80	118.44	134.06	25.03	0.17	-1.14	1.34	312.91	468.93	147.59	0.08	193.71	192.14	42.58	0.76	-0.08	1.66
total of Cortisol and Cortisone	631.11	894.32	258.62	0.76	681.69	808.32	115.61	0.20	-1.00	1.49	605.76	664.24	84.29	0.09	335.63	424.03	70.58	0.89	0.04	1.81
Total - all measured metabolites	845.31	1126.41	259.09	0.81	938.85	1052.30	126.68	0.17	-1.07	1.42	1070.78	1246.25	228.44	0.08	536.63	695.49	115.61	0.88	0.03	1.79
